# Assessment of the quality of effluent management from university hospitals in the Littoral department in Benin

**DOI:** 10.1186/s12889-021-11478-1

**Published:** 2021-07-19

**Authors:** Judicaël Todedji, Ghislain Sopoh, Cyriaque Degbey, Arouna Yessoufou, Fidèle Suanon, Daouda Mama

**Affiliations:** 1 Department of Environmental Health, Regional Institute of Public Health, University of Abomey-Calavi, Ouidah, Benin; 2 University Hospital Hygiene Clinic, National University Hospital Center Hubert Koutoukou Maga, Cotonou, Benin; 3 National Institute of Water, Laboratory of Applied Hydrology, University of Abomey-Calavi, Abomey-Calavi, Benin

**Keywords:** Assessment, Management, Effluents, CHU, Benin

## Abstract

**Background:**

Liquid discharges from hospitals (effluents) threaten the environment and are now a central concern of all stakeholders in the health system and those in the protection of the environment. The management of effluents is a major problem in developing countries. The objective of this study was to assess the quality of effluent management at the level of university hospital centers (CHU) in the Littoral region in Benin.

**Methods:**

It was a cross-sectional, descriptive, evaluative study that took place in 2020 to assess the “structure”, “process” and “results” components according to standard thresholds (Bad: < 60%; Acceptable: [60–80% [and Good: ≥ 80%).

**Results:**

In all the CHUs, all the components, as well as the overall quality of the management of hospital effluents, had a score between 0 and 60%, with an assessment deemed bad. The poor quality of the process highlighted the non-compliance with standards relating to the management of hospital liquid discharges. Several factors linked to the “structure”, “process” and “results” components at the same time explain this poor management of university hospitals effluents.

**Conclusion:**

These effluents discharged without prior treatment into wastewater could constitute a source of dissemination of potentially pathogenic microorganisms. It is therefore important to develop methods for treating these effluents before they are released into the natural environment.

## Introduction

Hospitals carry out a variety of activities for which various potentially toxic substances are used [1, 2]. For their operation, hospitals can consume a large amount of water per day, from 400 to 1200 l per day and therefore generate an equally volume of wastewater [3]. In addition to this high demand for drinking water, there is also the need for specific water such as physiological or sterilized water and serums [4]. Indeed, this significant water consumption by hospitals generates large volumes of liquid effluents loaded with pharmaceutical products, pathogenic microorganisms and chemical substances, which are generally discharged into urban networks without prior treatment, in the same way as conventional domestic wastewater [5]. The contact of this type of wastewater with the surrounding environment has harmful effects on the biological balance of aquatic ecosystems, causing an imbalance at different trophic levels [6]. Liquid hospital discharges threaten the environment much more than urban effluents and are today placed at the center of the concerns of all stakeholders in the health system and environmental protection [7, 8]. Poor management of hospital effluents could present a chemical, biological and physical risk to humans and their environment [9, 10]. These hospital effluents constitute a reservoir of potentially dangerous microorganisms capable of infecting hospital patients, health workers and the public when poorly managed [11]. They also contribute to the spread of multi-resistant germs in the environment. The presence of multi-resistant bacteria in the environment is a major public health problem. Hospital effluents have an ecotoxicity 5 to 15 times higher than that of urban effluents [12]. Apart from their therapeutic value, the medicinal substances contained in hospital effluents can interact with specific biological targets, which raises questions about the ecotoxicological and health risks associated with their presence in the environment [13]. Unlike macropollutants, micropollutants contained in hospital effluents can cause adverse effects in organisms at very low concentrations due to their toxicity, persistence and bioaccumulation.

In recent years, hospital effluents have received special attention in many countries around the world [14]. The management of biomedical waste has recently emerged as a subject of major concern not only for hospitals, health establishments, but also for structures in charge of environmental protection and law enforcement, the media and the general public [15]. In developed countries, hospital wastewater are collected by a sewerage network. This system transports this wastewater by pipes to the treatment plant (trickling filter, lagooning, activated sludge or biological discs) where it is treated before being released into the natural environment [16]. The latter makes it possible to purify wastewater and discharge the treated effluent which does not pose a danger to humans and their environment [17].

**Fig. 1:**
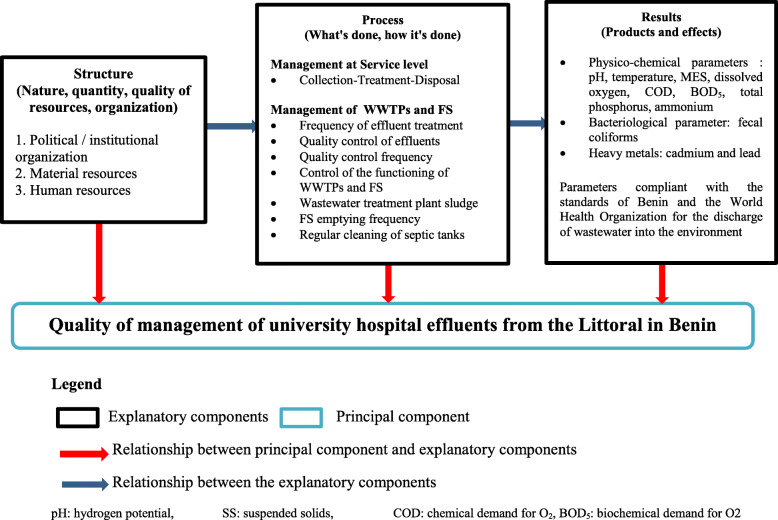
Conceptual framework of the quality of management of university hospital effluents from the Littoral in Benin

In developing countries, the management of liquid biomedical waste is a major problem with a lack of technology and skills to implement and monitor hospital waste management programs [18]. In these countries, biomedical waste is still handled and disposed of without discrimination, creating a huge threat to the health and the environment [19]. At the health facilities level, officials fail to install appropriate systems due to unavailability of technologies, insufficient financial resources and lack of professional training in biomedical waste management [15]. In Benin, the majority of hospitals do not have a wastewater treatment plant. This water is evacuated directly to the septic tanks (FS). Inadequate collection and treatment of sewage and sludge will result in the pollution of water sources with pathogens. It is therefore the ethical responsibility of the health facility management to be concerned with public health. In Benin, collection, transport and treatment of hospital effluents are major challenges for hospitals and health establishments. It is necessary to assess the current situation of the effluent management system and the general objective of this study was therefore to assess the quality of effluent management at the level of the university hospitals of the Littoral in Benin. The information generated is supposed to enable decision-makers at all levels to strengthen liquid biomedical waste management systems in order to improve the prevention and control of infections in health facilities in the country. As shown in Fig. 1, this study was carried out following the model of Donabedian in 2005, used not only to assess the quality of care, but also management in health structures [20]. According to this model, quality information can be classified at three levels: “structure”, “process” and “results”.

## Methods

### Study framework

**Fig. 2:**
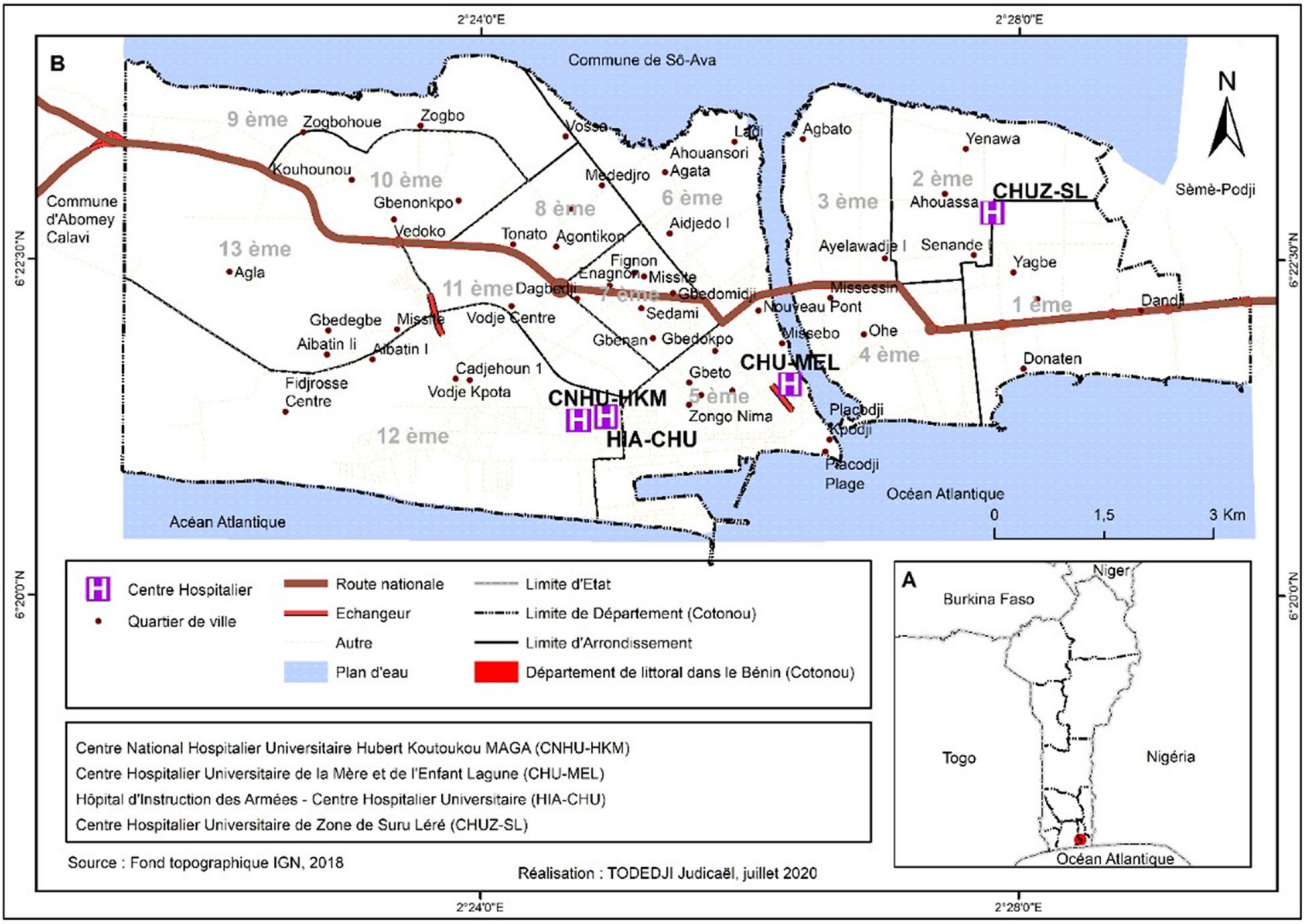
Location of university hospitals in the Littoral department

Our study was carried out in the university hospital centers (CHU) of the city of Cotonou and the station of the Industrial Company of Urban Equipment and Sanitation (SIBEAU). It was necessary to select the large university hospitals that cover a variety of disciplines, medical and medico-technical services and which offer a variety of pathologies cares. Four CHU met these criteria: The National University Hospital Center Hubert Koutoukou MAGA (CNHU-HKM), the University Hospital Center of Mother and Child Lagoon (CHU-MEL), the Army Instruction Hospital - University Hospital, (HIA-CHU) of Cotonou and the Zone University Hospital of Suru Léré (CHUZ-SL). The CNHU-HKM is the main health facility in Benin and has a competent medical team and several surgical and medical specialties to which are added the various specialties in diagnostic explorations. CHU-MEL is a benchmark hospital in the fields of pediatrics and gynecology-obstetrics. It includes the services of the child, the service of the mother and the medico-technical services. The main mission of the CHUZ-SL is to meet the health needs of the populations of the covered health zone and of patients from other health zones. It includes medical services and medico-technical services. The Cotonou HIA-CHU is under the supervision of the Directorate of Army Health Services, which reports directly to the Ministry of National Defense. It is a large referral hospital in the city of Cotonou. It has a good number of medico-technical services and a general administration. The SIBEAU treatment station is located in the district of Ekpè in the commune of Sèmè-podji and is the unique collective treatment station in Benin. The Fig. 2 shows the location of university hospitals in the Littoral department.

### Type and period of study

This was a cross-sectional, descriptive and evaluative study carried out over a period of 6 months from April to September 2020 on the quality of management of university hospitals effluents in the Littoral in Benin.

### Sampling

The study population consisted of staff involved in the management of hospital effluents. These included hygiene technicians, maintenance technicians, medical and radiology laboratory technicians, care assistants, maintenance officer and effluent samples. The sampling method for our study was non-probabilistic for all targets. Depending on the targets, the following techniques were used:

- Reasoned choice for the care services, the personnel of the hygiene and maintenance services and for the collection of effluents;
- Choice by convenience for medical and radiology laboratory staff and care assistants and maintenance officer.

The sample consisted of 04 hygiene technicians, 3 maintenance technicians, 11 laboratory technicians, 7 radiology technicians and 160 care assistants and maintenance officer for a total of 185 people. The effluent samples from the septic tanks of the CHU-MEL, the CHUZ-SL and the HIA-CHU were respectively 6, 8 and 12. One (01) sample was taken at the inlet and at the exit of the CNHU-HKM, CHU-MEL and SIBEAU wastewater treatment plants, i.e. three (03) at the entrance and three (03) at the exit to assess the efficacy of the treatment system. The samples taken at the SIBEAU treatment plant had made it possible to see whether the effluents from hospital septic tanks were discharged according to the legal standards. Samples for physico-chemical and toxicological analysis were taken in 1.5 l plastic bottles and those for bacteriological analysis were taken in 0.5 l glass bottles and transportation to laboratory.

### Definition of variables

The main variable of the study was the quality of university hospital effluent management which was explained by three components “structure”, “process” and “results”. For the operational aspect of the explanatory components, we used as a reference, the guidelines of the World Health Organization (WHO) of 2014 and standard thresholds for wastewater in the Republic of Benin of 2001 [21, 22]. Scores were calculated by simple weighting of each indicator to assess the quality of the management of hospital effluents. Descriptively, we considered that all indicators have the same weight. Thus, each criterion of each explanatory component was assigned a score of 0 for the “no” modality or a score of 1 for the “yes” modality. This defines the level of compliance or not with the standard. The criteria retained for each component are in Table 1.

**Table 1:** Expected score by criteria and components of the quality of effluent management at the level of each university hospital center on the Littoral in Benin

Criteria by component	Expected score
“Structure” component	
Human resources	**5**
Training on biomedical waste management	1
Continuing education in the management of liquid biomedical waste	1
Knowledge of the liquid biomedical waste management process	1
Knowledge of the causes of mismanagement	1
Knowledge of the risks generated by hospital effluents	1
Political / institutional organization	**11**
Existence of a Nosocomial Infections Control Committee (CLIN)	1
Existence of hospital hygiene teams	1
Functionality of CLIN	1
Involvement of the CLIN in the management of effluents	1
Existence of effluent management protocols	1
Existence of activity reports on effluent management	1
Existence of a monitoring plan for effluent management activities	1
Involvement of the hygiene department in the management of effluents	1
Existence of a maintenance plan for the WWTP and septic tanks	1
Designation of an effluent management manager	1
Existence of budget for wastewater management	1
Material resources	**5**
Availability of personal protective equipment (PPE)	1
Availability of washbasins	1
Availability of sump	1
Availability of infectious liquid waste collection containers	1
Availability of disinfectants	1
Subtotal 1	**21**
“Process” component	
Service level management	**9**
Sorting of effluents before pretreatment	1
Effluent pretreatment	1
Knowledge of the disinfectant used for pretreatment	1
Knowledge of the concentration of disinfectant used	1
Daily use of disinfectants	1
Respect of the contact time (at least 15 min)	1
Wearing personal protective equipment	1
Display of effluent management protocols	1
Display of effluent disinfection protocol	1
Management of WWTPs and Septic tanks (FS)	**7**
Effluent treatment frequency	1
Quality control of effluents	1
Quality control frequency	1
Control of the functioning of WWTPs and FS	1
Disposal of sludge from WWTP / FS	1
FS 1 oil change frequency	1
Maintenance and renewal of FS / STEP 1 treatment product	1
Subtotal 2	**16**
Component “Result”	
Physico-chemical characteristics	
Hydrogen potential [6-9]	1
Temperature < 30	1
Suspended Materials ≤35 *	1
Dissolved oxygen> 5 mg / L	1
Chemical O_2_ Demand (COD) < 125 *	1
Biochemical O_2_ demand (BOD_5_) < 25 *	1
Total phosphorus < 10	1
Ammonium < 0.2 mg / L	1
Bacteriological characteristics	
Faecal coliforms ≤2.10^3^	1
Toxicological characteristics	
Cadmium < 1 mg / L	1
Lead < 1 mg / L	1
Subtotal 3	**11**
Quality of management of university hospital effluents	
TOTAL (Subtotal 1+ Subtotal 2+ Subtotal 3)	**48**

In our study, for each criterion, was considered as a success an expected response or when more than 60% of respondents gave the expected response at the level of the “structure” and “process” component. An unexpected response or less than 60% of respondents gave the expected response was considered a failure. Analitically, the total scores obtained served as a basis for assessing each component. The expected score for each component is the following “structure” 21; “Process” 16 and “results” 11. Thus, a three-scale evaluation adapted with reference to the measurement scale of Varkevisser et al. [23] of the components and the quality of the management of hospital effluents would be: Good if the score percentage is between [80–100%]; Acceptable if the score percentage is between [60–80% [; Bad if the score percentage is less than 60%.

### Data collection and analysis

The data was collected using a questionnaire and by direct observation to relate and objectify the information collected during the questionnaires and observation within the services. Laboratory analyzes were carried out according to the protocols recommended by Rodier et al. [24] The parameters were analyzed in the Public Health Research and Expertise laboratory of the University Hospital Hygiene Clinic of the CNHU-HKM in Cotonou. To assess the efficacy of the WWTPs, the concentrations at the inlet were compared with those at the outlet. The collected data was encoded, computed and analyzed using IBM SPSS software, version 21. For quantitative variables, mean and standard deviation were calculated and for qualitative variables, percentages were calculated. The evaluation of the explanatory components and the quality of management of hospital effluents was carried out on the basis of the percentages of the scores obtained.

## Results

### Socio-professional characteristics of the interviewed hospital care assistants and maintenance officer

The average age of care assistants and maintenance officer involved in effluent management was 32 ± 7.53 years with extremes ranging from 18 to 54 years. They had an average professional seniority of 6 years ±5.9 (1–32 years). 50.6% of the respondents were between 25 and 35 years old and 60.6% had a professional seniority of less than 5 years. There were 1.19 women for 1 man. The majority of those surveyed had attained secondary education (68.8%). The majority of the care assistants and maintenance officer included in this study came from departments such as emergencies (14.4%), maternity (14.4%) and pediatrics (12.5%). These results are presented in Table 2.

**Table 2:** Characteristics of nursing assistants / maintenance workers

	Together (*n* = 160)	CNHU-HKM (*n* = 50)	CHU-MEL (*n* = 46)	HIA-CHU (*n* = 34)	CHUZ-SL (*n* = 30)
Age					
18–25 year	27 (16.9)	14 (28.0)	4 (8.7)	8 (23.5)	1 (3.3)
25–35 year	81 (50.6)	22 (44.0)	24 (52.2)	19 (55.9)	16 (53.4)
35–45 year	42 (26.2)	9 (18.0)	14 (30.4)	7 (20.6)	12 (40.0)
45 year and more	10 (6.3)	5 (10.0)	4 (8.7)	–	1 (3.3)
Sex					
Male	73 (45.6)	19 (38.0)	20 (43.5)	17 (50.0)	17 (56.7)
Feminine	87 (54.4)	31 (62.0)	26 (56.5)	17 (50.0)	13 (43.3)
Educational level					
Primary	25 (15.6)	2 (4.0)	6 (13.0)	14 (41.2)	3 (10.0)
Secondary	110 (68.8)	32 (64.0)	33 (71.8)	19 (55.9)	26 (86.7)
Superior	25 (15.6)	16 (32.0)	7 (15.2)	1 (2.9)	1 (3.3)
Professional seniority					
Less than 5 years	97 (60.6)	36 (72.0)	24 (52.2)	28 (82.4)	9 (30.0)
5–10 year	35 (21.9)	4 (8.0)	14 (30.5)	4 (11.7)	13 (43.4)
10–15 year	13 (8.1)	2 (4.0)	2 (4.3)	2 (5.9)	7 (23.3)
15 year and more	15 (9.4)	8 (16.0)	6 (13.0)	–	1 (3.3)
Services					
Emergency room	23 (14.4)	8 (16.0)	6 (13.0)	5 (14.7)	4 (13.3)
Maternity	23 (14.4)	8 (16.0)	5 (10.9)	5 (14.7)	6 (20.0)
Pediatrics	20 (12.5)	5 (10.0)	7 (15.2)	4 (11.8)	4 (13.3)
Operating room	9 (5.6)	–	4 (8.7)	3 (8.8)	2 (6.7)
Hospitalization	6 (3.7)	–	6 (13.0)	–	–
Surgery	18 (11.3)	7 (14.0)	4 (8.7)	4 (11.8)	3 (10.0)
Medical imaging	13 (8.1)	5 (10.0)	4 (8.7)	2 (5.9)	2 (6.7)
Medicine	14 (8.7)	6 (12.0)	-	5 (14.7)	3 (10.0)
Neonatology	19 (11.9)	5 (10.0)	5 (10.9)	4 (11.8)	4 (13.3)
Laboratory of medical biology	15 (9.4)	6 (12.0)	5 (10.9)	2 (5.9)	2 (6.7)

### Description of the “structure” component of effluent management quality

#### Competence of officers

More than half of the staff had received training in biomedical waste management. They were 60.6% in CHU-MEL and 80% in CHUZ-SL. Precisely 73.7% of the agents had not received any continuous training on the management of hospital effluents and this finding was the same at the level of the four hospitals in the study. Regarding the knowledge of the stages of management of hospital effluents, only 26.9% knew these stages. 60% of the agents had no knowledge of the causes of poor effluent management but 54.4% had knowledge of the risks associated with poor management of these effluents with a high rate at CNHU-HKM (60%).

#### Political / institutional organization

Only the CNHU-HKM has a nosocomial infection control committee (CLIN), all functional hospital hygiene teams and a protocol for managing hospital effluents. The CLIN was involved in the management of hospital effluents. No CHU produced activity reports on the management of hospital effluents and there was no monitoring plan for these management activities. The hygiene department was partially involved in the management of hospital effluents. All the four CHUs did not have a specific budget for the management of hospital effluents, a maintenance plan for the sanitation system (STEP and septic tanks). There was a manager in charge of effluent management at each hospital center.

#### Material resources

Personal protective equipment for maintenance workers was insufficient. At the service level, single-use personal protective equipment (PPE) such as masks (gloves, caps) and multi-use ones (gown, boots, apron) must be permanently available. The quantity of PPE available to healthcare services varies depending on the number of agents involved in the management of effluents. Observations and interviews show that the quantity of PPE available to services did not depend on the number of staff. At the CNHU-HKM, PPE such as masks and gloves were made available to care staff of services on a daily basis by the hospital pharmacy. Delays were sometimes observed in the provision of this PPE in the event of a shortage of stock.

The sinks and drains were insufficient in the observed services and the maintenance workers or orderlies had affirmed this.

Some services did not have a drain and the toilets reserved for patients were used as drains. There were insufficient collection containers of infectious liquid waste. The agents had said that the disinfectants used for decontamination were sometimes out of stock and the quantity provided by the hospital pharmacy was sometimes insufficient. This observation was the same at the level of the four university hospital centers.

### Description of the “process” component of effluent management quality

#### Service level management

In university hospital centers, waste sorting was not carried out in 51.9% of cases. 64.4% of workers did not pre-treat the waste before it was discharged into the sinks or drains. Almost three quarters of the staff knew about the disinfectant required for waste pretreatment but did not put this knowledge into practice. Most agents (78.1%) had no knowledge of the concentration of disinfectant used for waste pretreatment. Observations made in the services showed that no service had displayed the effluent management protocol as well as that of disinfection of hospital effluents. In the university hospitals that did not have a treatment station, the wastewater produced by the biomedical analysis and radiology laboratories was directly discharged into septic tanks or collecting basins serving as a receptacle for laboratory wastewater without any treatment through the drainpipes.

#### Management at the WWTP and septic tanks of hospital centers

The CNHU-HKM has an activated sludge wastewater treatment plant that operates only partially, so that the sludge produced is no longer discharged and remains at the bottom of the basin. The CHU-MEL has a biological disc treatment plant and also uses the septic tank system. Some hospital departments are connected to this treatment plant. The CHUs that do not have a WWTP such as the CHUZ-SL used a decentralized pretreatment system for wastewater, which is imposed due to the fact that the different services were not set up on the same day or the same period. This system was a three-compartment septic tanks equipped with sumps. These CHUs therefore had several septic tanks, at least one by department. In these CHUs, liquid waste was poured into the sinks or toilets of treatment rooms and drained to these septic tanks. The effluents produced by the CNHU-HKM and those evacuated to the CHU-MEL station are treated with chlorine. On the other hand, the effluents discharged to the septic tanks do not undergo any treatment once inside these tanks. The CNHU-HKM regularly checked the quality of these effluents despite the partial operation of its station. The quality control of the effluents treated by the CHU-MEL station had been carried out only once since its operation. The effluents discharged by the CNHU-HKM station are evacuated to the city gutters and those from the CHU-MEL are discharged into the Cotonou lagoon. The HIA-CHU and the CHUZ-SL had not carried out any quality control of the effluents produced. When the septic tanks were full, they were emptied by private emptying structures located throughout the country, which subsequently emptied this waste at the SIBEAU station.

#### Management in the SIBEAU treatment station

**Fig. 3:**
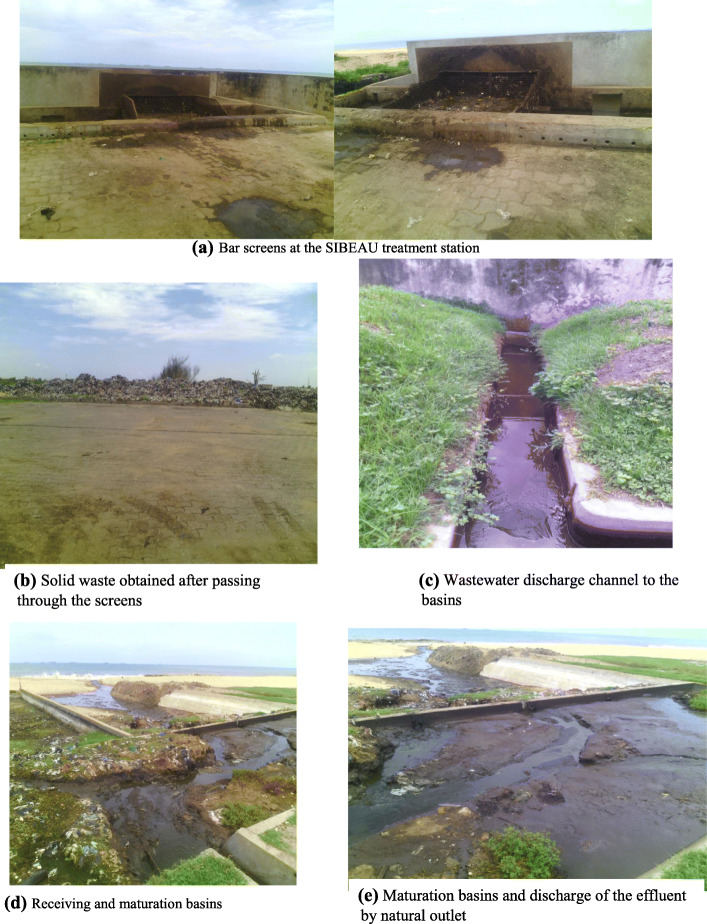
Management in hte SIBEAU treatment station

The SIBEAU treatment station is the only treatment station open to the public in Benin. It receives sludge from all the structures authorized by the cities of Cotonou, Sèmè Podji and Porto-Novo and their surroundings. The installations of the SIBEAU station include: the pretreatment equipment (two bar screens and a sand trap); treatment equipment (a reception basin, an optional basin and a maturation basin). Screening is the first level of treatment (pretreatment) where the raw effluent is stripped of its coarse elements. The ponds serve to purify both the liquid fraction coming from the sedimentation/thickening ponds. The effluent is discharged into the sea through a natural outlet. Today this station is not functioning properly due to its overload. Observations made on the site showed that the treatment system in place no longer meets the standards in the Republic of Benin (Fig. 3).

### Description of the “results” component of effluent management quality

The values of the pollution parameters (suspended solids, biochemical oxygen demand over 5 days, chemical oxygen demand, dissolved oxygen, ammonium, total phosphorus) of the effluents obtained at the outlet of the stations (after treatment) of the CNHU-HKM, the CHU-MEL and SIBEAU exceeded WHO and Benin standards for the discharge of wastewater into the environment (Table 3). The characteristics of the effluents were practically the same at the inlet and at the outlet except at the CHU-MEL where an average reduction of about 50% was noticed. The mean values of the effluent parameters from the septic tanks were very high compared to the standards of Benin and WHO.

**Table 3:** Physico-chemical parameters compared to Beninese and WHO standards

Parameters	CNHU-HKM		CHU-MEL			HIA-CHU	CHUZ SL	SIBEAU		Standards Benin/WHO
	I	O	I	O	FS	FS	FS	I	O	
pH	7.05	7.08	7.00	7.34	7.17	7.05	7.66	7.65	7.45	6–9
T (°C)	22.90	22.60	22.40	22.50	21.62	25.50	27.71	27.80	28.2	≤ 30
SS (mg/L)	93.00	80.00	111.00	68.00	65.67	105.17	109.63	9256.0	8230.0	≤ 35^a^
COD (mgO_2_/L)	424.00	289.00	271.71	143.85	150.18	256.33	41.01	6452.0	5230.0	≤ 125 ^a^
BOD_5_ (mgO_2_/L)	234.00	146.00	161.00	55.00	87.21	135.31	25.64	985.00	875.00	≤ 25 ^a^
DO (mg/L)	0.69	3.51	3.70	4.20	0.69	1.19	1.55	1.08	1.76	> 5
Am (mg/L)	9.36	2.10	23.76	7.28	55.11	90.02	68.19	102.0	93.0	< 0.2
PT (mg/L)	17.9	12.16	29.06	26.77	42.83	55.98	31.87	875.0	805.0	≤ 10

*I* Inlet, *O* Outlet, *FS* Septic tanks, *pH* potential of hydrogen, *T* Temperature, *SS* suspended matter, *COD* chemical demand in O_2_, *BOD*_*5*_ biochemical demand in O_2_, *DO* dissolved oxygen, *Am* Ammonium, *PT* total phosphorus

^a^Benin standard

The microbial loads of faecal coliforms in the effluents obtained at the outlet of the CNHU-HKM, CHU-MEL and SIBEAU stations (after treatment) and those of the effluents collected from the septic tanks were all above the standard of 2000 CFU/100 mL.

The concentrations of Lead and Cadmium in the effluents obtained at the outlet of the CNHU-HKM and CHU-MEL stations (after treatment) and those of the effluents collected from the septic tanks complied with the Benin standard for the discharge of wastewater into environment.

### Assessment of the different components and the quality of effluent management

The assessment of the different components was made from the scores obtained by the criteria defined. The scores obtained by criteria and then by component are presented in Table 4.

**Table 4:** Scores according to the criteria of the quality components of effluent management

Criteria		CNHU-HKM	CHU-MEL	HIA-CHU	CHUZ-SL
	ES	OS	OS	OS	OS
« Structure » Component					
Human resources	**5**	**1**	**1**	**0**	**1**
Training on biomedical waste management	1	0	1	0	1
Continuing education in the management of liquid biomedical waste	1	0	0	0	0
Knowledge of the liquid biomedical waste management process	1	0	0	0	0
Knowledge of the causes of mismanagement	1	0	0	0	0
Knowledge of the risks generated by hospital effluents	1	1	0	0	0
Political / institutional organization	**11**	**6**	**2**	**2**	**2**
Existence of a Nosocomial Infections Control Committee (CLIN)	1	1	0	0	0
Existence of hospital hygiene teams	1	1	0	0	0
Functionality of CLIN	1	1	0	0	0
Involvement of the CLIN in the management of effluents	1	0	0	0	0
Existence of effluent management protocols	1	1	0	0	0
Existence of activity reports on effluent management	1	0	0	0	0
Existence of a monitoring plan for effluent management activities	1	0	0	0	0
Involvement of the hygiene department in the management of effluents	1	1	1	1	1
Existence of a maintenance plan for the WWTP and septic tanks	1	0	0	0	0
Designation of an effluent management manager	1	1	1	1	1
Existence of budget for wastewater management	1	0	0	0	0
Material resources	**5**	**4**	**3**	**3**	**3**
Availability of personal protective equipment (PPE)	1	1	1	1	1
Availability of washbasins	1	1	1	1	1
Availability of sump	1	1	1	1	1
Availability of infectious liquid waste collection containers	1	0	0	0	0
Availability of disinfectants	1	1	1	1	1
“Process” component					
Service level management	**9**	**3**	**3**	**4**	**3**
Sorting of effluents before pretreatment	1	0	0	1	0
Effluent pretreatment	1	0	0	0	0
Knowledge of the disinfectant used for pretreatment	1	1	1	1	1
Knowledge of the concentration of disinfectant used	1	0	0	0	0
Daily use of disinfectants	1	1	1	1	1
Respect of the contact time (at least 15 min)	1	0	0	0	0
Wearing personal protective equipment	1	1	1	1	1
Display of effluent management protocols	1	0	0	0	0
Display of effluent disinfection protocol	1	0	0	0	0
Management of WWTPs and Septic tanks (FS)	**7**	**2**	**2**	**1**	**1**
Effluent treatment frequency	1	0	0	0	0
Quality control of effluents	1	1	1	0	0
Quality control frequency	1	1	0	0	0
Control of the functioning of WWTPs and FS	1	0	0	0	0
Disposal of sludge from WWTP / FS	1	0	0	0	0
FS 1 oil change frequency	1	–	1	1	1
Maintenance and renewal of FS / STEP 1 treatment product	1	0	0	0	0
“Result” Component					
Physico-chemical characteristics	**8**	**2**	**2**	**2**	**3**
Hydrogen potential [6-9]	1	1	1	1	1
Temperature < 30	1	1	1	1	1
Suspended Materials ≤35 *	1	0	0	0	0
Dissolved oxygen> 5 mg / L	1	0	0	0	0
Chemical O_2_ Demand (COD) < 125 *	1	0	0	0	1
Biochemical O_2_ demand (BOD_5_) < 25 *	1	0	0	0	0
Total phosphorus < 10	1	0	0	0	0
Ammonium < 0.2 mg / L	1	0	0	0	0
Bacteriological characteristics	**1**	**0**	**0**	**0**	**0**
Faecal coliforms ≤2.10^3^	1	0	0	0	0
Toxicological characteristics	**2**	**2**	**2**	**2**	**2**
Cadmium < 1 mg / L	1	1	1	1	1
Lead < 1 mg / L	1	1	1	1	1
Quality of management of university hospital effluents					
TOTAL	**48**	**20**	**15**	**14**	**14**

Legend: *ES* expected score, *OS* observed score

In all the CHUs, all the components as well as the overall quality of the management of hospital effluents had a score between [0–60%[, with an assessment deemed poor. The components were assessed from the total scores obtained and the quality of management by the total score of the components as shown in Table 5.

**Table 5:** Assessment of the components and the quality of the management of CHU effluents

Components		CNHU-HKM			CHU-MEL			HIA-CHU			CHUZ-SL		
	ES	OS	%	A	OS	%	A	OS	%	A	OS	%	A
Structure	21	11	52.4	BM	6	28.6	BM	5	23.8	BM	5	23.8	BM
Process	16	5	45.4	BM	5	31.2	BM	5	31.2	BM	4	25.0	BM
Results	11	2	26.6	BM	2	28.6	BM	2	28.6	BM	3	28.6	BM
Total	48	20	41.7	BM	15	31.2	BM	14	29.2	BM	14	29.2	BM

Legend: *ES* expected score, *OS* Observed Score, *A* Appreciation, % Percentage, *BM* Bad management

## Discussion

### Presentation of the main results of this work

The average age of maintenance personnel involved in effluent management was 32 ± 7.53 years with extremes ranging from 18 to 54 years. They had an average professional seniority of 6 years ±5.9 (1–32 years).

Seventy-three point seven percent of the workers had not received any continuous training in the management of hospital effluents. Regarding knowledge of the steps in the management of hospital effluents, only 26.9% were aware of these steps.

Only the CNHU-HKM has a nosocomial infection control committee, all functional hospital hygiene teams and a protocol for the management of hospital effluents. Personal protective equipment for maintenance workers was insufficient. At the university hospital level, waste sorting was not carried out in 51.9% of cases. 64.4% of workers did not pre-treat the waste before it was discharged into the sinks. Almost three quarters of the staff knew about the disinfectant required for waste pretreatment, but did not put this knowledge into practice.

The CNHU-HKM has a partially functioning activated sludge wastewater treatment plant. The CHU-MEL has a biological disc treatment plant and also uses the septic tank system. The effluents evacuated from the septic tanks are emptied at the level of the SIBEAU station. Observations made on the site showed that the treatment system of the SIBEAU station set up no longer meets the standards in force in the Republic of Benin.

The values of the pollution parameters were higher than the WHO and Benin standards for the discharge of wastewater into the environment.

In all the CHU, all the components as well as the overall quality of the management of hospital effluents had a score of between [0 and 60%]. We concluded that the quality of effluent management at each of these hospitals was poor.

### Quality and validity of the method used

The present study was descriptive and evaluative and took into account several university hospital centers. The quality of effluent management has been assessed at the level of each university hospital center. The non-probability sampling method followed by the reasoned choice and convenience technique allowed us to reach our targets and to constitute the size of our sample which is made up of staff involved in the management of hospital effluents at the level of hospital services. The choice of septic tanks and wastewater treatment plants sampled was made with the non-probabilistic method and the reasoned choice technique. It was a question of taking the septic tanks that receive effluents from services with the highest risk of infection and also the large tanks. The total number of agents involved in management was 180 for the four CHU and the number of samples taken was 32 for the four CHU. All the tools that have been developed in this study have been used as an excuse to ensure the consistency of the questions. Appropriate statistical tests were used to confirm the observed differences between the results obtained. We can confirm that the study has good method validity.

### Limitations of this study

Regarding the limits of the study, they are linked on the one hand to the non-generalizable character to all the hospitals of the Littoral department and of Benin in general because all the hospitals were not included in the study. Also, during this study, all the agents involved in the management of effluents at the level of the four hospitals were not surveyed and most were selected by the convenience technique. Certain biases were not lacking during the observation, in particular the presence of the observer, who must have influenced the behavior of professionals dealing with the management of infectious liquid waste at the service level. However, this state of affairs does not affect the quality of the recorded data. The administration of the data collection tools was carried out by ourselves; which allowed us to reduce the possible bias that could stain the understanding of our tools. Data collection took place in the four hospitals at different times. Information bias related to the administration of the tools was minimized by the choice of interviewers and by the triangulation of collection techniques. From the analysis of the above-mentioned provisions, we can say that our study results reflect the quality of the management of hospital effluents in the CHU of the Littoral department.

### Comparison of the main results obtained with those of other authors

#### Agent characteristics

In our study, more than half of the agents involved in the management of hospital effluents were women. This result was consistent with that of Assadulath et al. in 2013, and Kumar et al. in 2015 and contrary to the study of Bakobi et al. in 2018 [25–27], who reported that the majority of respondents (59%) of hospitals were men. The average age of the staff in our study was 32 years old. This result is similar to that of Deress et al. results in 2018 [28] who found an average age of 30.46 ± 6.64 years and Kumar et al. in 2015 [26], 31.15 years ±6.16 years. In our study, the average professional length was 6 years ±5.9 (1–32 years). Assadulath et al. [25] in 2013 has approximately reported the same duration of professional experience of 6.72 years.

#### Structure of hospital effluent management

The score obtained by the “structure” component in the four university hospital centers is less than 60%, therefore the quality of the structure was considered poor in the university hospitals. This poor quality noted is due to the poor quality of the human resources, the political/institutional organization and the material resources sub-components. The poor quality of the human resources sub-component is due to the fact that the majority of the agents involved in effluent management have not received any training in effluent management and they have a weak knowledge of the stages of effluent management and the health risks linked to poor management. A small number of personnel involved in the management of biomedical waste have received continuous training in the management of liquid biomedical waste. The training received took the form of retraining organized for the benefit of occasional agents recruited by the CNHU-HKM. For others, the training took the form of workshops and seminars. These results are in line with those of Deblij et al. in 2019 [29] in Maroc who stated that effluent managers received little continuous training in this area. Sawalem et al. in 2009 [30] in Libya showed that 85% of waste management staff were not trained in hospital waste management and had insufficient knowledge of potential hazards. Training is a prerequisite for the establishment of an appropriate management system and is a determinant of good management of hospital waste. Indeed, all staff involved in effluent management should be trained to understand the benefits of the healthcare waste management system and the responsibilities that will be involved [31]. However, in the hospitals involved in the study, only the CNHU-HKM has a functional CLIN. Regarding the political/institutional organization, in terms of hospital hygiene service (SHH), only the CNHU-HKM has a university hospital hygiene clinic. The other CHUs have a hygiene and sanitation service instead of a hospital hygiene service. However, these services try to play the role of hospital hygiene service and are partially involved in the management of hospital effluents. According to the recommendations of the World Health Organization [21], each healthcare establishment should draw up a simple management plan for waste from healthcare activities setting out the objectives, activities, stakeholders and their responsibilities, the necessary resources, as well as monitoring, supervision and control mechanisms. There is no standard procedure (technical guides or guidelines; control procedures) for the collection, transport, storage and treatment of infectious liquid waste in the study hospitals. The same observation was made by Sanogo et al. in 2014 [32] in two university hospitals in Bamako in Mali and by Ahmed et al. [33] in Sudan who indicated that out of a large number of hospitals, only 25% have a documented policy describing the management of medical waste. The absence of the reference document on the management of hospital effluents was noted in the majority of CHUs. The low availability of reference documents on the management of hospital waste in the hospitals of the study is explained by the absence of the CLIN, responsible for developing the internal policy for the management of biomedical waste and making it available to the care services and ensure its application by all stakeholders in the health structure. In all the CHUs visited, there is no specific budget dedicated to the management of hospital liquid waste. One could say that there is no political will regarding the management of liquid biomedical waste. All resources are focused on the management of solid biomedical waste, resulting in staff neglect which is reflected in the results of the management of this liquid biomedical waste.

The various problems linked to the effluent management structure were mainly the non-existence of hospital hygiene promotion structures (hospital hygiene service, hospital hygiene unit, hospital hygiene operational team), the insufficient qualified human resources, the absence of a management plan for liquid biomedical waste, the insufficient quantity of PPE and a specific budget for the management of liquid biomedical waste. All these problems could be explained by the failure to apply the document of the national hospital hygiene policy in the Republic of Benin. Each hospital should be inspired by this national policy document to develop its internal hospital hygiene policy document which will contribute to the good management of liquid biomedical waste within the hospital. Solving these problems involves developing and implementing internal hospital hygiene policy and hospital effluent management protocols. The managers of these CHU should ensure that part of the financial resources of hospitals are devoted to the management of liquid biomedical waste.

#### Hospital effluent management process

The process of managing hospital effluents in the four CHUs had obtained a score of less than 60%. This score confirms the score of the political/institutional organization sub-component of the structure. There is presence and use of disinfectant in the departments observed, but note that compliance with the dilution procedure, the daily use of the disinfectant, and compliance with the contact time were not effective. The steps in the management of hospital effluents were not followed. A minority of the agents involved in management knew the process but did not put this knowledge into practice. This could be explained by the ignorance of the agents or by the ignorance of the health and environmental risks linked to the mismanagement of hospital effluents. The lack of training, monitoring and reference documents on the management of hospital effluents could also explain these behaviors.

Almost three quarters of the staff knew about the disinfectant required for waste pretreatment but did not put this knowledge into practice.

Wearing personal protective equipment before handling infectious liquid waste was not systematic in the departments we visited. This could be explained by the insufficiency or shortage of stock of these small materials for individual use at the service level. The septic tanks are functional in the CHU and the emptying is done regularly but the quality control of the effluent discharged from these tanks is not done. This constitutes a danger for the surrounding population of the spillway site. It should also be noted that the product (coal) in the tanks as a treatment product is not renewed after emptying. These coals serve to absorb organic substances from wastewater. However, there is the phenomenon of saturation which makes these products ineffective after a certain time so they must be renewed regularly. Despite Decree No. 2006–087 of March 8, 2006 [34] approving the national hospital hygiene policy document in the Republic of Benin, which stipulates that a preliminary treatment be carried out before the discharge of liquid waste into septic tanks, sumps and into the environment, hospital effluents do not undergo any treatment before discharge into septic tanks and sumps. This decree is not being applied because it is unknown to health officials and professionals. The effluent quality control is carried out regularly by the CNHU-HKM despite the partial operation of its station. In Italy, Spain and Germany, pretreatment of hospital effluents is not compulsory locally. In France, community laws, especially for infectious diseases [35], recommend disinfection. In Iran, hospitals are obliged to pretreat their wastewater themselves. In addition, in these hospitals, the waste is not discharged into the municipal drain networks. Since then, each hospital has managed its rejections by using a treatment department, which is affiliated to it, and which reuses them [36]; especially since recently, these effluents may have aroused the interest of researchers who have expressed concern about their chemical, radiological and carcinogenic risks [37]. In our study, septic tanks are emptied by private structures located across the country when they are full. These structures will then empty these effluents at the level of the SIBEAU treatment station. The observations made at the station showed that the SIBEAU station no longer meets the standards of the treatment of faecal sludge. These results are in accordance with the program completion report of the African Development Bank (Benin) of 2017 [38]. This report stated that the only SIBEAU wastewater treatment plant which is operational throughout the Grand Nokoué area, constituted of the communes of Porto-Novo, Sèmè-Podji and Cotonou, is in disrepair because more than 2/3 of the structure has been destroyed by marine erosion. Spilled sludge only passes through the rest of the structures before ending up at sea without treatment [38].

From the analysis of the results, it emerges that the problems related to the hospital effluent management process were the insufficient wearing of personal protective equipment, the non treatment of effluents before discharge into the environment and the non-existence of management protocols of hospital effluents. This state of affairs could be explained by the fact that the texts and laws on the management of biomedical waste are too general in scope and there is no legal framework specifically focused on liquid biomedical waste. To resolve these various problems, the managers of these university hospitals should create hospital hygiene services in hospitals where these services are non-existent, establish a real policy for the management of hospital effluents and give them the same interest as the solid biomedical waste and build hospital wastewater treatment and purification plants. The effluent management equipment must be made available on a permanent basis to the agents involved and all care and services personnel must receive continuous training in the management of liquid biomedical waste.

#### Result of the management of hospital effluents

The result of the management of hospital effluents in the four CHUs had obtained a score of less than 60%. This score is due to the fact that the pollution parameters obtained after treatment at the wastewater treatment plants and septic tanks did not comply with the standards for discharging wastewater into the environment apart from pH and temperature. The pH results presented in this study showed that these effluents have an alkaline pH. This result is consistent with Bouzid et al. result in 2013 [39]. The discharge of effluents with a total phosphorus value higher than the standard in surface water could lead to eutrophication [40]. The concentration of suspended solids is higher than that set by WHO [41] and their degradation consumes a significant amount of available oxygen. Therefore, the photosynthetic process of phytoplankton underwater will decrease due to the limited dissolved oxygen in the aquatic environment [42]. The analysis of the results shows that the BOD_5_ and COD concentrations are very high. The average quantity of fecal coliforms found in all the University Hospital of the Littoral department was higher than the standards. The effluents are very loaded with pollutants and constitute a threat to the environment and to health. The results of the analysis had shown that the characteristics of the wastewater were the same at the inlet and outlet of the SIBEAU treatment station. However, this station receives in addition to sludge from domestic septic tanks, the sludge from hospital septic tanks using the decentralized wastewater pretreatment system. Therefore, hospital wastewater is discharged into the sea without any treatment. Since 2002, the study carried out within the framework of the “sustainable management of waste and urban sanitation” program [43] had reported that the SIBEAU station does not function properly because of its overload and the efficacy of station is poor. The effluents discharged have COD and BOD_5_ characteristics close to those of the sludge brought by the trucks. Up to 3600 mg/L of BOD_5_ pass through the basins before being discharged into the sea located less than 10 m [44].

To contribute to the good management of the effluents produced by the CHU involved in this study, a report of the results obtained at the end of this study will be made to the authorities of each hospital center together with proposals for improvement.

## Conclusion

The management of hospital effluents is a very important issue in hospitals and healthcare facilities. The results of our study showed that the effluents produced by the university hospitals of the Littoral are not well managed. The management of liquid discharges in Benin does not meet the legal standards. Faced with all these problems, the rehabilitation of WWTPs is essential. This study therefore represents a preliminary step in building a database for subsequent studies on effluent management at university hospital level. These effluents discharged without prior treatment into surface water could constitute a source of dissemination of potentially pathogenic germs. It is therefore important to develop methods for treating these effluents before they are released into the natural environment. For effective implementation of effluent management practices in hospitals, a periodic awareness and continuing education program is mandatory to improve knowledge and practices of liquid biomedical waste management among healthcare workers.

## Data Availability

The data presented in this study are available on request from the corresponding author.

## Abbreviations

BOD: Biochemical O_2_ demand
CHU: University Hospital Centers
CHU-MEL: University Hospital Center of Mother and Child Lagoon
CNHU-HKM: National University Hospital Center Hubert Koutoukou MAGA
CHUZ-SL: Zone University Hospital Center of Suru Léré
CLIN: Nosocomial Infections Control Committee
COD: Chemical O_2_ Demand
FS: Septic Tanks
HIA-CHU: Army Instruction Hospital - University Hospital Center
PPE: Personal Protective Equipment
SHH: Hospital Hygiene Service
SIBEAU: Station of the Beninese Industrial Company of Urban Equipment and Sanitation
WHO: World Health Organization
